# Verifying a questionnaire diagnosis of asthma in children using health claims data

**DOI:** 10.1186/1471-2466-11-52

**Published:** 2011-11-22

**Authors:** Connie L Yang, Teresa To, Richard G Foty, David M Stieb, Sharon D Dell

**Affiliations:** 1 Division of Respiratory Medicine, Hospital for Sick Children, Toronto, Canada; 2 Child Health Evaluative Sciences, Hospital for Sick Children, Toronto, Canada; 3 Health Canada, Ottawa, Ontario, Canada

## Abstract

**Background:**

Childhood asthma prevalence is widely measured by parental proxy report of physician-diagnosed asthma in questionnaires. Our objective was to validate this measure in a North American population.

**Methods:**

The 2884 study participants were a subsample of 5619 school children aged 5 to 9 years from 231 schools participating in the Toronto Child Health Evaluation Questionnaire study in 2006. We compared agreement between "questionnaire diagnosis" and a previously validated "health claims data diagnosis". Sensitivity, specificity and kappa were calculated for the questionnaire diagnosis using the health claims diagnosis as the reference standard.

**Results:**

Prevalence of asthma was 15.7% by questionnaire and 21.4% by health claims data. Questionnaire diagnosis was insensitive (59.0%) but specific (95.9%) for asthma. When children with asthma-related symptoms were excluded, the sensitivity increased (83.6%), and specificity remained high (93.6%).

**Conclusions:**

Our results show that parental report of asthma by questionnaire has low sensitivity but high specificity as an asthma prevalence measure. In addition, children with "asthma-related symptoms" may represent a large fraction of under-diagnosed asthma and they should be excluded from the inception cohort for risk factor studies.

## Background

Parental proxy report of physician-diagnosed asthma (questionnaire diagnosis) is the standard measure of childhood asthma prevalence used in national health surveys and epidemiological studies. The International Study of Allergies and Asthma in Childhood (ISAAC) questionnaire is the current gold standard for ascertaining asthma outcomes in epidemiologic studies [1]. The "ever wheeze" question is often used to compare asthma prevalence between countries. For international studies, a symptom-based definition is less subject to bias than a diagnosis-based definition[2]. However, for national studies that evaluate risk factors, a more specific definition is often desired [3-5]. In these studies, lifetime asthma is measured by affirmative response to the question: "Has your child ever had asthma?" and is further defined to be "Doctor diagnosed asthma" if there is an affirmative response to the question "Has this been diagnosed by a doctor?" Despite its widespread use in studies assessing asthma prevalence[2], risk factors [6],and diagnostic tests [7] this questionnaire based asthma diagnosis has not been validated in a North American population.

Canada has a universal health care system in which all Canadians have equal access to physician and hospital services. Health claims data on all patient encounters is collected for administrative purposes although in Ontario there is currently no centralized database on prescription medication. These databases have been found to be accurate compared to chart abstraction for diseases such as ischemic heart disease[8], esophagitis[9] as well as asthma[10]. Recently, an algorithm has been developed to identify children with asthma from health claims data in the province of Ontario. This algorithm, has been shown to have 91.4% sensitivity and 82.9% specificity for correctly identifying asthma when compared to expert consensus diagnosis of asthma[10,11].

The objective of this research was to assess agreement between questionnaire based parental proxy report of physician diagnosed asthma in children (hereafter referred to as "questionnaire diagnosis") and asthma diagnosed by analyzing health claims data (hereafter referred to as "health claims diagnosis") in a population-based sample of urban Canadian school children.

## Methods

### Subjects

Participants were recruited from The Toronto Child Health Evaluation Questionnaire (T-CHEQ) study. This study used ISAAC methodology[1] to recruit a population based sample of 5619 children (aged 5 to 9 years) from 231 Toronto public schools between January and May of 2006. The demographic characteristics of these children closely resembled census data[12] and the prevalence of asthma outcomes closely resembled national health survey data[12]. Detailed methods for sampling and recruitment are published elsewhere[12]. All subjects that participated in the T-CHEQ study were asked at the time of the initial study if they would consent to have their child's questionnaire linked to health claims data for research purposes and those that agreed were included in this study.

### Study Design

This is a diagnostic validation study, comparing agreement between questionnaire asthma diagnosis (using data from the cross-sectional T-CHEQ study) and health claims diagnosis (using cohort data from lifetime health claims administrative databases).

### Questionnaire based Asthma diagnosis

Asthma diagnosis was identified from the T-CHEQ study sample by affirmative responses to the questions "Has your child ever had asthma?" and "Was this diagnosed by a doctor?" Those that reported non-physician diagnosed asthma were excluded from the analysis. Non-asthma controls did not report doctor diagnosed asthma. These controls were further categorized into those with "Asthma-related symptoms" if they reported a yes response to either "Has your child ever had wheezing" or "In the past 12 months has your child had a dry cough at night, apart from a cough associated with a cold or chest infection?".

### Health Claims Asthma Diagnosis (Reference Standard)

Health claims data between March 31, 1997 and March 31, 2006 (i.e. the child's lifetime) from two Ontario health care administrative databases were used: (1) the Canadian Institute for Health Information (CIHI) discharge abstract database for inpatient services and (2) The Ontario Health Insurance Plan (OHIP) for ambulatory and emergency services. Both of these databases contain diagnostic codes based on the International Classification of Disease (ICD)-9 or 10. A claim for asthma was identified by the ICD- 9 code 493 for claims up to March 31, 2002 and ICD-10 codes J45 and J46 identified asthma in subsequent years. The CIHI database currently allows up to 25 diagnostic codes (prior to 2002, 16 diagnostic codes were allowed) and if any of these was for asthma, the hospitalization was included. The OHIP database allows one diagnostic code per visit. Only one claim per physician per day per patient was allowed. The health claims databases were also linked to the Registered Persons Database which contains mortality and demographic data to ensure that the subjects had lived within the province since birth. A unique personal identifier (the scrambled Health Card Number) included in each database permits the linkage of a child's records across all databases and time while preserving patient confidentiality.

Prevalent asthma cases were defined by a previously validated algorithm as follows: at least one hospitalization for asthma at any time during the child's life or two separate ambulatory or emergency room visits for asthma within a two year time frame[11]. This algorithm was previously found to have optimal diagnostic parameters using an expert consensus diagnosis from chart abstraction[10,11].

### Data linkage

The T-CHEQ participants were anonymously linked to the health claims databases through their reported Health Insurance Number. A matching date of birth in the two databases was also needed for the link to be considered valid. All data linkage and analysis related to this study was completed within the secure confines of the Institute of Clinical and Evaluative Sciences in Toronto, Ontario.

### Statistical methods

"Questionnaire diagnosis" and "health claims diagnosis" were compared in two by two tables. Sensitivity and specificity of the questionnaire diagnosis were calculated using health claims diagnosis as the gold standard. In order to test the potential misclassification bias that could occur by including children with asthma-related symptoms in the non-asthma control group, the children with asthma-related symptoms were removed from the sample and sensitivities and specificities were recalculated. Additional sensitivity analyses were conducted by modifying the health claims data algorithm (increasing the time frame for incident asthma ambulatory claims from 2 to 3 years or including emergency visit data from an additional database). We also calculated agreement (kappa)[13] between questionnaire and health claims diagnosis.

### Ethical Approval

Parents of children in this study gave informed consent for participation in this research by filling in the voluntary T-CHEQ questionnaire and additionally agreeing to participate in the data linkage. This study was approved by the Research Ethics Board of the Hospital for Sick Children in Toronto.

## Results

### Baseline characteristics

From the original TCHEQ cohort, 2884 (51.32%) gave permission to link to health claims data. Respondents did not differ from non-respondents in terms of asthma prevalence or gender, however they were more likely to be in a higher income group (42.13% versus 31.40%) and have post-graduate education (51.96% versus 40.09%), and were less likely to report no physician visits in the past year (24.85% versus 29.57%) (Table 1). Data linkage was successfully achieved in 2782 of the respondents (96.46%).

**Table 1:** Characteristics of Toronto School Children with Asthma Questionnaire and Health Claims Data, 2006^a^

Variable	Respondents n = 2884 n (%)	Non-respondents n = 2735 n (%)	*P*-value
Physician Diagnosis of asthma (n = 5461)	438 (15.71%)	409 (15.34%)	0.73
Male sex (n = 5573)	1459 (50.84%)	1313 (48.58%)	0.09
Mean age in years (n = 4781)	6.75	6.72	0.20
Income adequacy (n = 5241)			<0.0001
Lowest income	469 (16.82%)	466 (19.00%)	
Lower middle	568 (20.37%)	592 (24.14%)	
Upper middle	577 (20.69%)	624 (25.45%)	
Highest income	1175 (42.13%)	770 (31.40%)	
Highest level of education (n = 5421)			<0.0001
Less than high school	189 (6.75%)	267 (10.19%)	
Completed high school	278 (9.92%)	340 (12.98%)	
Completed postsecondary education	879 (31.37%)	962 (36.73%)	
Completed post graduate education	1456 (51.96%)	1050 (40.09%)	
Number of visits to health care provider in the last year (n = 5237)			<0.0001
0	678 (24.85%)	742 (29.57%)	
1-4	1471 (53.92%)	1213 (48.35%)	
>=5	579 (21.22%)	554 (22.08%)	
Physician visits in last year (n = 5237)			<0.0001
No regular physician	678 (24.85%)	742 (29.57%)	
Pediatrician only	936 (34.31%)	771 (30.73%)	
General Practitioner only	486 (17.82%)	375 (14.95%)	
Pediatrician & General Practitioner	628 (23.02%)	621 (24.75%)	
Environmental tobacco smoke exposure in home (n = 5383)	302 (10.94%)	328 (12.51%)	0.07

^a^Respondents are T-CHEQ participants who agreed to health claims data linkage while non-respondents did not agree and are therefore not included in the study population. The T-CHEQ study was a population-based sample.

### Asthma prevalence

In our study sample, 437 children were identified with questionnaire diagnosis of asthma (prevalence of 15.71%) while 586 children were identified with health claims diagnosis of asthma (prevalence of 21.06%) (Table 2). The vast majority of subjects with ever asthma (defined by the question "Has your child ever had asthma?") had physician diagnosed asthma (also responded affirmatively to the question "Was this diagnosed by a doctor?") (prevalence of asthma 16.41% and 15.71% respectively).

**Table 2:** Questionnaire^a ^Asthma Diagnosis versus Health Claims^b ^Diagnosis (n = 2782)

	**Health Claims **+	Health Claims -	Total
Questionnaire +	346	91	437
Questionnaire -	240	2105	2345
Total	586	2196	2782

^a ^Positive response to questions "Has your child ever had asthma?" and "Was this diagnosed by a physician?"

^b^Algorithm of one asthma hospitalization and/or two asthma physician visit claims within two years

Kappa = 0.60 (95% CI 0.56, 0.64)

Sensitivity = 59.04% Specificity = 95.86% (Health Claims reference standard)

### Questionnaire accuracy

The questionnaire diagnosis had a sensitivity of 59.04% and a specificity of 95.86% for detecting asthma using the health claims diagnosis as the reference standard.

Of the 2435 non-asthma controls, 854 (35%) had asthma-related symptoms. When these children were removed from the sample (table 3), the sensitivity and specificity of the questionnaire diagnosis were 83.57% and 93.56% respectively.

**Table 3:** Questionnaire^a ^Asthma Diagnosis versus Health Claims^b ^Diagnosis, excluding children with "asthma-related symptoms" (n = 1826)

	Health Claims +	Health Claims -	Total
Questionnaire +	346	91	437
Questionnaire -	68	1321	1389
Total	414	1412	1826

^a ^Positive answer to questions "Has your child ever had asthma?" and "Was this diagnosed by a physician?"

^b ^Algorithm of one asthma hospitalization and/or two asthma physician visit claims within two years

Kappa = 0.75 (95% CI = 0.72, 0.79)

Sensitivity = 83.57% Specificity = 93.56% (Health Claims Reference Standard)

The sensitivity analysis performed using modified algorithms for definitions of health claims diagnosis did not produce any significantly different findings (data not shown).

We observed moderate agreement (kappa = 0.60) between questionnaire asthma definitions and health claims asthma definitions. Good agreement (kappa = 0.75) was observed when those with asthma-related symptoms were excluded.

## Discussion

Our findings concur with other literature that suggests that questionnaire asthma diagnosis is specific but not sensitive for asthma[14-17]. As expected, compared with questionnaires that use a definition of "wheezing in the last twelve months" to define the population with asthma, our definition of physician diagnosed asthma was more specific but less sensitive[18]. In epidemiologic studies that estimate prevalence, a highly sensitive test is preferable; whereas, studies that estimate risk require more specific tests [19].

Excluding children with asthma-related symptoms from the sample increased the sensitivity (from 59% to 84%) and overall agreement (kappa increased from 0.60 to 0.75) between questionnaire and health claims diagnoses. Children with asthma-related symptoms may represent a substantial proportion of under-diagnosed asthma. This may have implications for cohort studies producing risk estimates[20] for putative risk factors for asthma incidence. Our findings support the practice of excluding children with asthma-related symptoms from the control group in epidemiological studies in order to decrease misclassification bias[20]. A limitation to this approach is the inflation of the odds ratio that occurs and the divergence of the odds ratio from the relative risk, making it impossible to calculate population-attributable risk from an exposure.

The diagnosis of asthma is problematic as subjects are often asymptomatic with normal physical examinations and normal pulmonary function tests between exacerbations[21]. This problem is compounded in children as they are often unable to do pulmonary function testing would might help to clarify the diagnosis. As such, the diagnosis often relies on symptom report which is subject to significant recall bias [22]. Given these limitations, health claims databases are a useful source of information as they capture data at the time of asthma exacerbation.

A larger issue in the study of asthma is that there is no accepted gold standard to confirm the diagnosis; therefore, studies evaluating diagnostic tests must use an imperfect reference standard. The accuracy of the test being evaluated is a measure of how closely it correlates with the reference standard. Given that the questionnaire and the health claims diagnosis measure different aspects of physician-diagnosed asthma, it is not surprising that the questionnaire has good validity against the health claims reference standard.

We have capitalized on the population-based data available through our universal health care system to validate the questionnaire asthma diagnosis in our T-CHEQ population. The results of this study may not be generalizable to a population that does not have equal access to health care.

This study is however the largest validation study reported to date and gives evidence that parental report on questionnaire is a highly specific method for identifying children with asthma in Canada.

## Conclusions

Parental proxy report of asthma diagnosis by questionnaire has low sensitivity but high specificity as an asthma prevalence measure for epidemiological studies. Excluding children with asthma-related symptoms from non-asthma control groups will result in less misclassification bias.

## Abbreviations

ISAAC: International Study of Allergies and Asthma in Childhood; TCHEQ: Toronto Child Health Evaluative Questionnaire; CIHI: Canadian Institute for Health Information; OHIP: Ontario Health Insurance Plan; ICD: International Classification of Disease.

## Competing interests

The authors declare that they have no competing interests.

## Authors' contributions

SDD, TT, DS conceived of the study and participated in its design and coordination. RF cleaned and prepared data for linkage procedures. CLY performed the literature review and prepared the first draft of the manuscript. All authors read and approved of the manuscript.

## Pre-publication history

The pre-publication history for this paper can be accessed here:

http://www.biomedcentral.com/1471-2466/11/52/prepub
